# Predicting Unmet Healthcare Needs in Post-Disaster: A Machine Learning Approach

**DOI:** 10.3390/ijerph20196817

**Published:** 2023-09-24

**Authors:** Hyun Jin Han, Hae Sun Suh

**Affiliations:** 1 Department of Regulatory Science, Graduate School, Kyung Hee University, Seoul 02447, Republic of Korea; hyunjin.han@khu.ac.kr; 2 Institute of Regulatory Innovation through Science (IRIS), Kyung Hee University, Seoul 02447, Republic of Korea; 3 College of Pharmacy, Kyung Hee University, Seoul 02447, Republic of Korea

**Keywords:** supervised machine learning, post-disaster management, healthcare utilization

## Abstract

Unmet healthcare needs in the aftermath of disasters can significantly impede recovery efforts and exacerbate health disparities among the affected communities. This study aims to assess and predict such needs, develop an accurate predictive model, and identify the key influencing factors. Data from the 2017 Long-term Survey on the Change of Life of Disaster Victims in South Korea were analyzed using machine learning techniques, including logistic regression, C5.0 tree-based model, and random forest. The features were selected based on Andersen’s health behavior model and disaster-related factors. Among 1659 participants, 31.5% experienced unmet healthcare needs after a disaster. The random forest algorithm exhibited the best performance in terms of precision, accuracy, Under the Receiver Operating Characteristic (AUC-ROC), and F-1 scores. Subjective health status, disaster-related diseases or injuries, and residential area have emerged as crucial factors predicting unmet healthcare needs. These findings emphasize the vulnerability of disaster-affected populations and highlight the value of machine learning in post-disaster management policies for decision-making.

## 1. Introduction

Disasters exacerbate or even catalyze existing disparities in health and healthcare access among affected populations [1,2,3,4]. Unfulfilled healthcare needs after a disaster are critical factors that delay recovery, worsen health outcomes, and exacerbate health disparities in the aftermath of disasters [5,6,7]. In the event of a disaster, there is a higher demand for health resources compared to their usual capacity, but the resources may be scarce due to fragmented health infrastructure and the failure of healthcare continuity [1,6,8]. This phenomenon is especially distressing for vulnerable populations struggling with persistent healthcare disparities and limited post-disaster healthcare access [9,10,11]. 

Unmet healthcare needs refer to the limited access to healthcare resources or insufficient service provision [12,13,14]. The World Health Organization (WHO) emphasizes the importance of ensuring access to healthcare not based on affordability but needs for health promotion [15] because unmet healthcare needs affect health outcomes [16,17]. In previous studies, patients who failed to meet their healthcare needs experienced functional diminishment or disease progression [7,18]. Unmet healthcare needs are used as a complementary measure of health inequity [14,19], posing an undeniable challenge in post-disaster recovery. Medically under-served communities are particularly vulnerable to disasters, and there is ample evidence that unmet healthcare needs after a disaster deteriorate the health status of disaster victims [1,20,21,22,23,24,25].

The Sendai Framework for Disaster Risk Reduction 2015–2030 (Sendai Framework) emphasizes the importance of assessing health outcomes of disasters and prioritizing vulnerable populations [26]. Consequently, identifying unmet healthcare needs among the affected populations and predicting vulnerability post-disaster have become crucial policy objectives in post-disaster management [5]. However, to the best of our knowledge, research on the development of predictive algorithms for unmet healthcare needs in post-disaster settings and management is lacking. Aligned with the Sendai Framework, post-disaster management revolves around assessment and prediction because it enables improved resource allocation. However, the complex and dynamic nature of disasters and post-disaster conditions pose challenges in understanding and predicting the unmet needs of affected individuals. Moreover, while the disaster management field seeks technological advancements for more accurate estimation and prediction [27,28,29,30,31], there is currently no existing contextual analysis that incorporates diverse post-disaster experiences influencing unmet healthcare needs, nor any prediction models for such needs. Using a descriptive analysis of specific disaster cases, previous studies have examined factors such as ethnic differences, gender, underlying health conditions, and income that impact unmet healthcare needs following a disaster [5,7]. However, these studies were unable to provide predictions regarding populations with unmet healthcare needs or generalize the factors that influence unmet healthcare needs.

This study addresses the limitations of previous research by estimating the unmet healthcare needs in a post-disaster context and developing a predictive algorithm for such needs using a supervised machine learning approach. The objective is to enhance post-disaster management through more accurate prediction and understanding of unmet healthcare needs. Additionally, this study explores the key factors that contribute to the prediction of unmet healthcare needs based on the developed predictive model.

## 2. Materials and Methods

### 2.1. Data Source 

The data for this study were obtained from the 2017 Long-term Survey on Life Changes of Disaster Victims (LSCLDV), a representative population-based survey of disaster victims in South Korea. The LSCLDV, a disaster victim panel survey, was first designed for the long-term investigation of natural or social disaster victims and the development of relief policy and technology in South Korea by the National Disaster Management Research Institute (NDMRI). Beneficiaries of the government disaster relief fund from 2012 are registered in the National Disaster Management System (NDMS). Registered households were contacted, and 47% of the contacted households consented and agreed to be surveyed by the NDMRI to determine the number of family members and eligible adult respondents. Stratified nationwide sampling was conducted based on this population and the survey was launched in 2016. The survey questionnaires included core question sets regarding disaster victims’ social status, disaster experience, disaster relief services, and funding experiences, along with additional questionnaires tailored to the specific interests of each survey year. Detailed descriptions of the questionnaires, respondents’ uptake policies, and datasets are available on the NDMRI website (https://www.ndmi.go.kr/ (accessed on 10 August 2022)) [32]. This study was approved by the Institutional Review Board of Kyung Hee University (KHSIRB-22-270(EA)). The requirement for informed consent was waived by the IRB because secondary and deidentified data were used for the analyses.

### 2.2. Measurement of Unmet Healthcare Needs

Unmet healthcare needs were measured using a dichotomous dependent variable based on the 2017 LSCLDV. The 2017 survey is the only year in which unmet healthcare needs related to disaster illnesses were specifically examined. Respondents were asked about proper healthcare service provision for disaster-related illnesses or injuries. Those who reported being “Not served” or “Needed but not served timely” were considered to have experienced unmet healthcare needs. 

### 2.3. Variables for Prediction Algorithms

To ensure computational efficiency and logical coherence, relevant and explainable features were selected from the LSCLDV dataset, whereas irrelevant features were discarded to reduce the computational burden [33]. The feature selection process was guided by Andersen and Newman’s health behavior model, which is a well-established framework for understanding medical resource utilization [34]. The model comprises three domains: predisposing factors, enabling factors, and need factors. 

**Figure 1:**
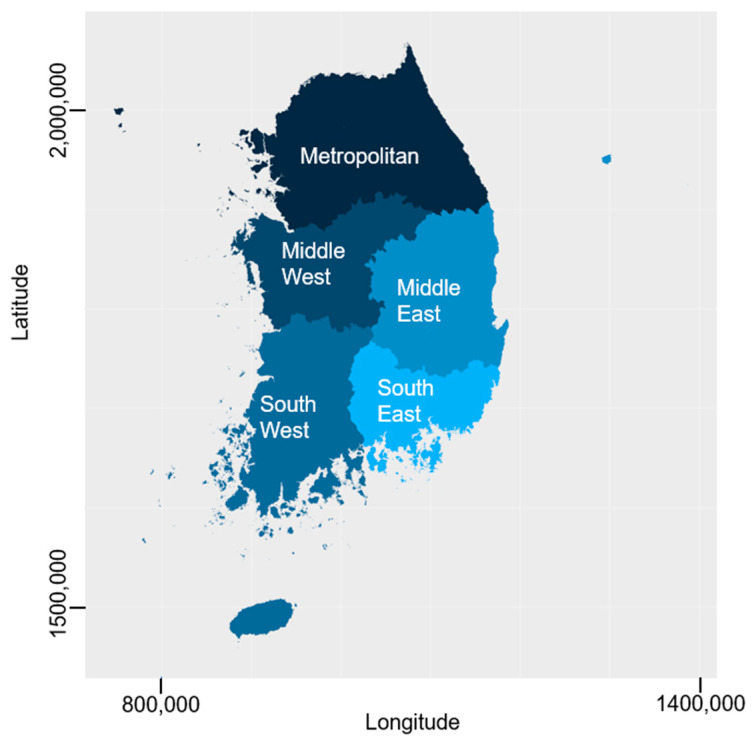
Four disaster territories for South Korea’s disaster management.

Predisposing factors include variables such as sex, age, marital status, and educational level. These characteristics provide insights into the demographic and social profiles of individuals accessing healthcare services. Enabling factors include variables related to the resources available to individuals, families, or communities. In this study, household income and residential area were considered enabling factors. Household income reflects the financial resources to which individuals have access, whereas residential areas serve as proxies for community-level resources. Residential areas were coded based on government-defined disaster territories for effective disaster management, as shown in Figure 1. The four territories were defined according to their geographical locations as follows: The Metropolitan territory (Seoul, Incheon, Gyeonggi, and Gangwon), the Midwest territory (Daejeon, Sejong, Chungbuk, and Chungnam), the Southwest territory (Gwangju, Jeonbuk, Jeonnam, and Jeju), the Mideast territory (Daegu and Gyeongbuk), and the Southeast territory (Busan, Ulsan, and Gyeongnam).

Meanwhile, the needs factor domain focuses on perceived healthcare needs, including variables such as perceived health status, chronic disease status, physical disability, and symptoms of depression. These factors provide an understanding of an individual’s health conditions and the perceived need for healthcare services [35].

Furthermore, considering the impact of disasters on individual lives, variables related to disaster events and subsequent life changes were included in the dataset. These variables include the type of disaster experienced, income changes after the disaster, changes in debt status, and whether individuals received subsidies after the disaster.

### 2.4. Machine Learning and Statistical Analyses

In this study, variables were described using numbers and percentages for both categorical and continuous variables. To estimate disaster-related unmet healthcare needs, statistical tests were performed to determine whether there were significant differences based on factors related to health resource utilization. For continuous variables, t-tests were conducted with a 95% confidence interval. This test allowed us to compare the means of two groups to assess whether there was a significant difference between them.

There are only a few Machine Learning (ML) methods studies on unmet healthcare needs. However, recent studies have used decision-tree algorithms to predict unmet healthcare needs. For example, the unmet mental health treatment needs of adults in the United States were predicted using a hierarchical optimal discriminant analysis with a tree classifier. Similarly, South Korean adults’ unmet dental care needs and their associated factors have also been explored using decision tree algorithms [36,37]. Although ML methods offer a better predictive performance, their lack of interpretability can hinder their usefulness in informing public health policies and interventions. Understanding the factors and mechanisms underlying unmet healthcare needs is essential for developing effective interventions for health promotion. Interpretable models such as decision trees provide a clearer understanding of the relationships between predictors and outcomes, making them valuable in public health policy [38,39].

This study utilized supervised ML techniques, specifically logistic regression, C5.0, and random forest models, to circumvent existing limitations. These models excel in their interpretability compared to their counterparts, thereby facilitating a deeper comprehension of the factors tied to unmet healthcare needs. This understanding is invaluable for deriving relevant insights into the implications of public health policy [40]. Despite being traditional, logistic regression remains a frequently used method for modeling binary-dependent variables. Its performance is further enhanced through ML, which automates feature selection from the data and thereby optimizes the logistic regression model [41]. C5.0, and random forest models, which are both tree-based algorithms, offer advantages such as managing various variable types, handling missing values, and providing superior interpretability. In addition, decision trees exhibit a distinct advantage in handling categorical variables, thereby serving as a valuable analytical instrument for dissecting the complex, multi-dimensional data sets frequently seen in public health studies [42]. Owing to its rule-based classification technique, C5.0, a low-memory classifier, exhibits high accuracy and efficiency. This technique uses ‘if-then’ condition rules extracted from dataset attributes and class labels. An unpruned tree is initially created in this rule-based model, followed by rule application to each path for reduction using algorithmically determined thresholds. This postprocessing phase permits new rule creation, effectively capturing vital characteristics from previous rule data while discarding irrelevant classifications [43,44]. Random forest, a multiple-decision tree aggregator, demonstrated excellent predictive performance. Several studies have affirmed its superiority over traditional decision tree algorithms and highlighted the significance of variable importance in predictions. Additionally, this algorithm is particularly effective for handling high-dimensional data and identifying complex interactions between features [45]. This makes random forest a widely used algorithm across many fields, including disaster studies, even with the emergence of advanced ML techniques [46,47,48].

In this study, we developed a supervised deep learning algorithm that systematically splits the data into training and testing sets and progressively establishes and compares predictive models. We allocated 75% of the total data set for training and 25% for testing. This split was based on a learning curve analysis to determine an appropriate training data volume to achieve reliable results. For the logistic regression, we identified the binary variable of unmet needs as the dependent variable (y) and predicted the likelihood of occurrence using independent variables. A stepwise backward logistic regression was performed on the training dataset to select the optimal model, with the Akaike Information Criterion (AIC) value guiding the selection. The predictive power of the model was then assessed using the test dataset by comparing the Area Under the Receiver Operating Characteristic (AUC-ROC) value and the misclassification rate. The C5.0 algorithm was implemented using the C50 library in R, setting the minimum number of nodes to 10 and number of iterations to 100, following a specific pruning rule. These hyperparameters were carefully chosen to optimize the model’s predictive power while also ensuring computational efficiency. The Random Forest model was analyzed using the Random Forest package, applying the Leave-Group-Out Cross Validation (LGOCV) methodology for model training and test dataset resampling. For hyperparameter tuning, we employed a grid search approach combined with cross validation. This method systematically explored a range of values for critical hyperparameters such as the number of trees in the forest, the number of features to consider for each split, and the minimum number of samples required to split an internal node. Through this exhaustive search, we ensured that our model was not only accurate but also computationally efficient.

**Figure 2:**
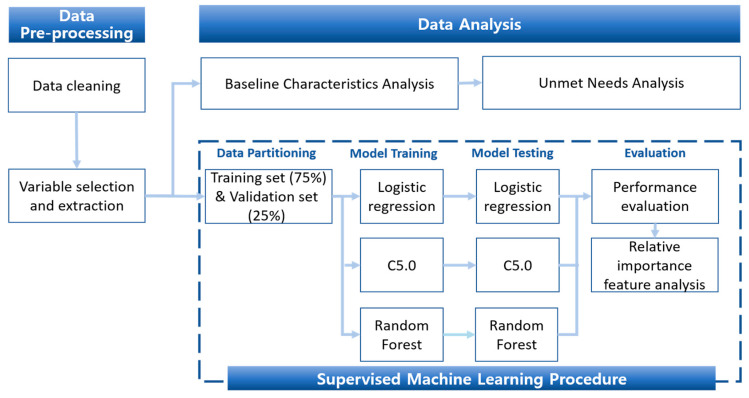
Data analysis and machine learning process.

We assessed the prediction performance of the models using metrics such as the precision, accuracy, misclassification rate, AUC-ROC value, and F-1 score. The variable importance of most predictive models was investigated using the mean decrease in accuracy and Gini score methods. The analysis process is illustrated in Figure 2. All statistical analyses were carried out using R x64 version 3.6.3 (http://www.R-project.org/ (accessed on 2 March 2020)), R Foundation for Statistical Computing).

## 3. Results

### 3.1. Study Population

Our study included 1659 adult respondents from the LSCLDV 2017 survey, aged between 13 and over 65 years, with a mean age of 58.3 (±17.7) years. The sex distribution was slightly skewed towards females (853, 51.4%). A substantial number of respondents were married (1129 or 68.1%), and approximately half (845 or 50.9%) had an education level of high-school or above. The majority (1188 or 71.6%) reported an income within the range of 1–5 million Korean won.

Most participants resided in the southwestern and southeastern areas, accounting for 1009 individuals (60.9%). Mean self-rated health score was 4.4 (±1.4). Chronic disease was reported by 31.8% (*n* = 527) of the respondents, and disabilities were reported by a small fraction (0.5% or *n* = 9). Depression was common among respondents, with 59.2% (*n* = 959) experiencing it.

Natural disasters were the primary type of disaster encountered, affecting 93.4% of the sample (1549 individuals). Typhoons were the most prevalent, accounting for 49.01% of the sample, followed by heavy precipitation at 35.20% and earthquakes at 9.16%. Post-disaster financial effects varied: 25.5% (423 individuals) reported a decrease in income, 17.1% (284 individuals) experienced an increase in debt, and a smaller group (3% or 49 individuals) received government subsidies.

### 3.2. Estimating Unmet Healthcare Needs Related to Disasters

Table 1 presents the characteristics of the participants based on their post-disaster unmet healthcare needs. Among all participants, 523 (31.5%) experienced unmet healthcare needs after the disaster. There were significant differences in all variables when comparing the group with unmet healthcare needs to those without.

Regarding demographic characteristics, the group with unmet healthcare needs had a higher proportion of females, elderly, and divorced/separated/widowed individuals, as well as a lower education level. Health status variables, including self-rated health scores, perceived chronic disease, disability, depression, and disaster-related injuries or disease incidents, were significantly worse among those with unmet healthcare needs.

The average self-rated health score was significantly lower among those with unmet healthcare needs (3.77 ± 1.4) compared to those without (4.62 ± 1.4). The rate of perceived chronic disease was significantly higher in the unmet needs group (216, 41.3%) than in the group without unmet healthcare needs (311, 27.4%). Depression was prevalent among participants, but with a higher occurrence in the unmet healthcare needs group (374, 71.5%) than in the group without unmet healthcare needs. The incidence of disaster-related injuries or diseases was also significantly higher among those with unmet healthcare needs (114 or 21.8%) than among those without them (29, 2.6%).

Finally, the type of disaster experienced differed significantly between the groups. For example, those with unmet healthcare needs encountered social disasters more frequently (60 or 11.5%) than those without them (50 or 4.4%).

### 3.3. Predictive Model for Unmet Healthcare Needs

Three supervised ML models were used to determine the best predictive model. A machine learning algorithm cannot ensure perpetual performance, and its performance can vary according to the features related to the datasets, methods, and goals of the analysis [49].

The performance of each trained model was assessed using a validation dataset, with detailed results, including the precision, accuracy, misclassification rate, AUC-ROC, and F-1 score (Table 2). According to the AUC-ROC (0.83) and F-1 scores (76.51%), the random forest algorithm demonstrated the best performance in predicting unmet healthcare needs among disaster victims, outperforming both logistic regression and C5.0 algorithms.

### 3.4. Important Variables in the Models

Following the performance test results, we examined the relative importance of individual variables contributing to the prediction of unmet healthcare needs among disaster victims using the best-performing random forest model. Variable importance was assessed using the mean decrease in accuracy plots and Gini scores ranked in descending order. The mean decrease in accuracy reflects the impact of variables on classification accuracy, whereas the mean decrease in the Gini coefficient measures each variable’s contribution to node and leaf homogeneity within the random forest model. Thus, a higher mean decrease in accuracy or Gini score signifies greater variable importance in the model.

**Figure 3:**
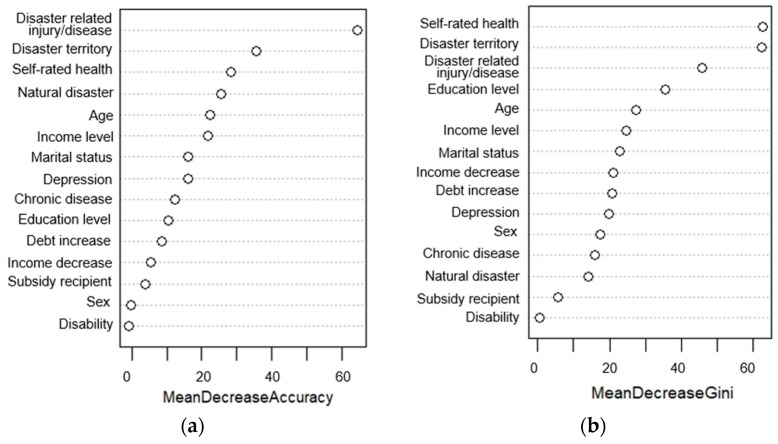
Relative important factors of unmet healthcare needs: (**a**) Mean Decrease Accuracy; (**b**) Mean Decrease Gini.

Figure 3 shows the relative importance and ranking of the variables for predicting unmet healthcare needs. Of the 15 factors incorporated into the model, disaster-related injury or disease, residential region in relation to the disaster territory, and self-rated health were deemed the most critical factors both in the context of accuracy and Gini score. Disability was consistently the least significant variable. Notably, exposure to natural disasters was the fourth most important factor for model accuracy but was among the least influential variables for model homogeneity.

## 4. Discussion

To the best of our knowledge, this is the first study to address the estimation of unmet healthcare needs in a nationally representative sample of disaster victims and to use machine learning to develop a predictive model for these needs. The significance of predicting unmet healthcare needs for post-disaster management and recovery underscores the importance of our work from both the disaster policy and public health perspectives.

Previous studies have reported increased unmet healthcare needs in disaster-experienced populations compared to the general public, which is in line with our own findings. Here, we estimated unmet healthcare needs using a dataset from LSCLDV. We found that 31.5% of post-disaster victims experienced unmet healthcare needs and had a significantly lower self-related health status (3.8 scores) compared to those without unmet healthcare needs (4.6 scores) (*p* < 0.000). Thus, our results show that disaster-exposed populations suffer from more unmet healthcare needs than the general population or other vulnerable populations in South Korea.

Despite the medical aid provided to disaster victims in South Korea, our study revealed that these individuals continue to face significant unmet healthcare needs, exceeding the rates seen in the general population. Prior research has estimated that the percentage of unmet healthcare needs is 11–17% among the general population, 19.7% among community-dwelling disabled individuals, 20.9% among individuals with physical disabilities or brain lesions, and 23.6% among the elderly in South Korea [50,51,52]. Notably, the burden of out-of-pocket (OOP) healthcare expenses in South Korea is relatively higher than that in other member countries of the Organization for Economic Co-operation and Development (OECD) [53,54,55,56]. The high rate of OOP expenses is often identified as the primary reason for unmet healthcare needs in South Korea, even in the context of universal health insurance [57,58,59]. In response, the Korean government provides medical aid to disaster victims, waiving OOP expenses for six months post-disaster when the victims’ residential areas are designated as special disaster zones due to severe impact. Despite being eligible for this benefit, our study population continued to experience higher rates of unmet healthcare needs compared to the general Korean population.

The prediction and management of unmet healthcare needs following disasters is vital for effective recovery and a return to normalcy. Although there is broad agreement on the importance of predictive technologies for disaster prevention and post-disaster management, there is a need for more research focusing on public health. Our study addresses this gap by pioneering a predictive model that marries public health behavioral theory with advanced supervised machine learning methodologies. We employed a meticulous feature selection and analysis process, aiming for unrivaled predictive performance. The resulting model, leveraging the random forest algorithm, exhibits exemplary predictive capabilities, outperforming in metrics such as precision, accuracy, misclassification error, AUC-ROC, and F-1 scores [60]. These results align with but exceed the performance of traditional methodologies. Our findings provide valuable insights into the field of post-disaster management and underscore the utility of machine-learning approaches for decision-making in public health post-disaster management.

Regardless of the method of analysis, the three most crucial factors associated with unmet healthcare needs following disasters were self-rated health, disaster-related injury or disease, and residential regions within government-designated disaster territories. These findings highlight the critical roles of health status and residential location in post-disaster public health planning. Our findings have several important implications for future studies. First, they underscore the ongoing need for policy support to ensure adequate healthcare resources for the vulnerable in the post-disaster recovery process. Numerous studies have corroborated the significant influence of health status on healthcare utilization, emphasizing its relevance for unmet healthcare needs among disaster-affected populations [7,61,62]. Second, our findings highlight the importance of addressing the intersection of regional and socioeconomic vulnerabilities with disaster susceptibility as a strategy to reduce unmet healthcare needs after disasters. The impact of a disaster is a product of the disaster event itself and regional variables. Regions often associated with both high disaster vulnerability and limited accessibility to healthcare resources are typically characterized by low-income, rural communities, and pre-existing vulnerabilities [1,8,63].

Previous research has thoroughly examined the relationship between disaster impacts and individual demographic characteristics such as gender, age, ethnicity, and social class. Socially disadvantaged populations have lower disaster preparedness and recovery levels, emphasizing the need for post-disaster management to address these disparities [5,64]. Our study acknowledges the significance of demographic characteristics in predicting post-disaster unmet healthcare needs. However, in the context of disaster scenarios in South Korea, these demographic factors appear to be less critical compared to health conditions and region of residence. Therefore, to effectively address unmet healthcare needs following a disaster, policy development should primarily focus on individuals’ health conditions and the specific regional characteristics of disaster victims.

This study has several limitations. First, the analysis relied on cross-sectional data from a disaster victim panel from a single year (2017), and so caution should be applied when considering the generalizability of the results. Despite this, the data remain valuable due to the diverse composition of disaster victims over a five-year span (2012–2016) and the provision of information across various post-disaster periods. The Long-term Survey on the Change of Life of Disaster Victims (LSCLDV), which is a representative sample of national disaster victims, aids in validating the population’s generalizability. Second, the self-reported nature of the survey presents a constraint in terms of the clinical accuracy of the diagnoses. We attempted to mitigate this by incorporating various health indicators into our analysis such as self-rated health scores, disaster-related diseases and injury status, depression measures, chronic diseases, and injury status. Third, including 13-year-old individuals possibly causes age-related bias in the education level factor. Our dataset includes only two subjects who are 13 years old, and neither of these participants exhibited any unmet healthcare needs. Given their limited number and absence of unmet healthcare needs, the potential for age-related bias impacting our model’s unmet healthcare need predictive accuracy and reliability is minimal. Fourth, it should be noted that the heterogeneity in disaster exposure levels and the systemic barriers constraining healthcare access—such as distance to healthcare facilities and sociocultural marginalization—constitute limitations of the present study that are not accounted for in the predictive algorithm. While our model does include critical variables like geographical location and income tiers, we recognize their likely inadequacy in comprehensively capturing the multi-faceted factors affecting healthcare accessibility in disaster scenarios. Finally, owing to data limitations, we could not account for disease severity, which is a crucial factor in determining healthcare needs within the predictive model. However, we indirectly considered disease severity through the inclusion of self-rated health status scores in the predictive model, which, in turn, influenced the need for healthcare resources.

## 5. Conclusions

This study confirms that individuals exposed to disasters are at increased risk of unmet healthcare needs compared to the general population. Our research contributed to a significant research gap by employing machine learning to predict these unmet needs across various disaster scenarios. Notably, our predictive model serves as a practical tool for shaping more targeted post-disaster management policies that better address the needs of vulnerable groups. For the future research agenda, there is a need for extensive research exploring the various phenomena and predictions concerning unmet healthcare needs.

## Figures and Tables

**Table 1:** Demographic characteristics and post-disaster unmet healthcare needs.

Variables	Total	Unmet Healthcare Need		*p*-Value
		No	Yes	
*N*	1659	1136	523	
Gender				<0.000 *
Male	806 (48.6)	595 (52.4)	211 (40.3)	
Female	853 (51.4)	541 (47.6)	312 (59.7)	
Age (in years)				<0.000 *
13–18	41 (2.5)	39 (3.4)	2 (0.4)	
19–39	222 (13.7)	169 (14.9)	53 (10.1)	
40–64	725 (44.0)	525 (46.2)	200 (38.2)	
65 and over	671 (32.7)	403 (35.5)	268 (51.2)	
Marital status				<0.000 *
Unmarried	252 (15.2)	198 (17.4)	54 (10.3)	
Married	1129 (68.1)	779 (68.6)	350 (66.9)	
Divorced, separated, or widowed	278 (16.8)	159 (14.0)	119 (22.8)	
Education level				<0.000 *
Elementary school or less	517 (31.2)	313 (27.6)	204 (39.0)	
Middle school	297 (17.9)	195 (17.2)	102 (19.5)	
High school	619 (37.3)	447 (39.3)	172 (32.9)	
University or higher	226 (13.6)	181 (15.9)	45 (8.6)	
Monthly household income (KRW)				<0.000 *
Less than 1 M	341 (20.6)	196 (17.3)	145 (27.7)	
1 M~less than 5 M	1188 (71.6)	838 (73.8)	350 (66.9)	
5 M~less than 9 M	120 (7.2)	94 (8.3)	26 (5.0)	
9 M or higher	10 (0.6)	8 (0.7)	−0.4	
Residential area				<0.000 *
Metropolitan	248 (14.9)	176 (15.5)	72 (13.8)	
Middle west	215 (13)	155 (13.6)	60 (11.5)	
South west	550 (33.2)	403 (35.5)	147 (28.1)	
Middle east	187 (11.3)	86 (7.6)	101 (19.3)	
South east	459 (27.7)	316 (27.8)	143 (27.3)	
Self-rated heath, mean (SD)	4.4 (1.4)	4.6 (1.4)	3.8 (1.4)	<0.000 †
Chronic disease				<0.000 *
Yes	527 (31.8)	311 (27.4)	216 (41.3)	
No	1132 (68.2)	825 (72.6)	307 (58.7)	
Disability				0.023 *
Yes	9 (0.5)	3 (0.3)	6 (1.1)	
No	1650 (99.5)	1133 (99.7)	517 (98.9)	
Depression				<0.000 *
Yes	959 (59.2)	585 (51.5)	374 (71.5)	
No	660 (40.8)	551 (48.5)	149 (28.5)	
Type of disaster experience				<0.000 *
Natural disaster	1549 (93.4)	1086 (95.6)	463 (88.5)	
Social disaster	110 (6.6)	50 (4.4)	60 (11.5)	

Notes: Statistical analysis: * chi-squared test, † unpaired Student *t*-test. Abbreviations: KRW, Korea Won; SD, standard deviation.

**Table 2:** Predictive performance of post-disaster unmet healthcare needs prediction models.

	Precision	Accuracy	Misclassification Rate	AUC-ROC ^1^	F-1 Score
Logistics regression	70.00%	75.20%	24.8%	0.774	70.59%
C5.0	65.40%	76.60%	17.1%	0.805	73.62%
Random forest	79.20%	77.59%	23.71%	0.830	76.51%

^1^ AUC-ROC = area under the receiver operating characteristic curve.

## Data Availability

The datasets generated and analyzed during this study are available, at any time, upon request at www.ndmi.go.kr (accessed 25 April 2022).
